# An Improved Deep Forest Model for Predicting Self-Interacting Proteins From Protein Sequence Using Wavelet Transformation

**DOI:** 10.3389/fgene.2019.00090

**Published:** 2019-03-01

**Authors:** Zhan-Heng Chen, Li-Ping Li, Zhou He, Ji-Ren Zhou, Yangming Li, Leon Wong

**Affiliations:** ^1^The Xinjiang Technical Institute of Physics and Chemistry, Chinese Academy of Sciences, Ürümqi, China; ^2^University of Chinese Academy of Sciences, Beijing, China; ^3^College of Engineering and Applied Science, University of Colorado Boulder, Boulder, CO, United States; ^4^ECTET, Rochester Institute of Technology, Rochester, NY, United States

**Keywords:** self-interacting proteins, disease, position-specific scoring matrix, deep learning, wavelet transform

## Abstract

Self-interacting proteins (SIPs), whose more than two identities can interact with each other, play significant roles in the understanding of cellular process and cell functions. Although a number of experimental methods have been designed to detect the SIPs, they remain to be extremely time-consuming, expensive, and challenging even nowadays. Therefore, there is an urgent need to develop the computational methods for predicting SIPs. In this study, we propose a deep forest based predictor for accurate prediction of SIPs using protein sequence information. More specifically, a novel feature representation method, which integrate position-specific scoring matrix (PSSM) with wavelet transform, is introduced. To evaluate the performance of the proposed method, cross-validation tests are performed on two widely used benchmark datasets. The experimental results show that the proposed model achieved high accuracies of 95.43 and 93.65% on *human* and *yeast* datasets, respectively. The AUC value for evaluating the performance of the proposed method was also reported. The AUC value for *yeast* and *human* datasets are 0.9203 and 0.9586, respectively. To further show the advantage of the proposed method, it is compared with several existing methods. The results demonstrate that the proposed model is better than other SIPs prediction methods. This work can offer an effective architecture to biologists in detecting new SIPs.

## Introduction

Proteins, highly complex substance, are the main compound of all the life. It is also the material basis and the first element of the life. Individual proteins rarely works in isolation. Most of proteins can work together with molecular partners or other proteins, which are associated with protein-protein interactions (PPIs) (Chou and Cai, 2006; You et al., 2014b,c; Li et al., 2017). One special case of PPIs is self-interacting proteins (SIPs), whose more than two identities can interact with each other to form a homodimer or homotrimer or homo-oligomer (Marianayagam et al., 2004), play key roles in the understanding of celluar process and cell functions. These interactions have received much more attention than they have done in recent years. Ispolatov et al. (2005) specified that the quantity of SIPs is more than twice as much as that of other proteins in the protein interaction network (PIN) (You et al., 2010a, 2014a, 2015b, 2017c; Liu et al., 2013; Huang et al., 2016a; Li et al., 2016), which point out the function of SIPs importance for cellular systems, so as to better understand the effect of disease mechanism. Pérez-Bercoff et al. (2010) considered that the genes of SIPs may have higher duplicability than others, and their research focus on the whole-genome level rather than the small scale. Hashimoto et al. (2011) presented several molecular mechanisms of self-interaction, mainly includes ligand-induced, domain swapping, insertions, and deletions. As a result, most previous works focus on the individual SIPs with the level of structures and functions. To our current knowledge, there are a great deal of computational techniques based on machine learning and deep learning (Gui et al., 2009; You et al., 2010b, 2015a, 2017a,b; Lu et al., 2013; Mi et al., 2013; Huang et al., 2015; Chen et al., 2016, 2018a,b,c; Gui et al., 2016; Huang et al., 2016b; Li et al., 2018) which applied in the field of bioinformatics and genomics, in which they were few for detecting protein interactions.

Recently, Zhou et al. (2012) developed a PPI model for PPIs prediction, which inputs condon pair frequency difference into a support vector machine (SVM) predictor. Particularly, You et al. (2013) presented a novel method which combined principal component analysis (PCA) with ensemble extreme learning machine model to predict PPIs based on the amino acid sequences information. Since the proposed feature extraction method has a higher discriminative power to reveal most of the information from protein sequences, they are great success for PPIs detection. Zahiri et al. (2013) introduced a PPIevo algorithm based on evolutionary feature which extracted from position-specific scoring matrix (PSSM) of known protein sequences. Du et al. (2014) designed a predictor for SIPs by applying random forest with the ensemble coding method, which integrated many biochemical properties and useful features. Zhang et al. (2018) predicted PPIs by using a ensemble deep neural networks (DNN) based on various of representations of protein sequences. Li et al. (2017) detected the SIPs based on evolutionary information and amino acids sequences by using ensemble learning method. Although these methods were relatively mature for PPIs prediction, there were few machine learning and deep learning methods to predict SIPs.

**FIGURE 1:**
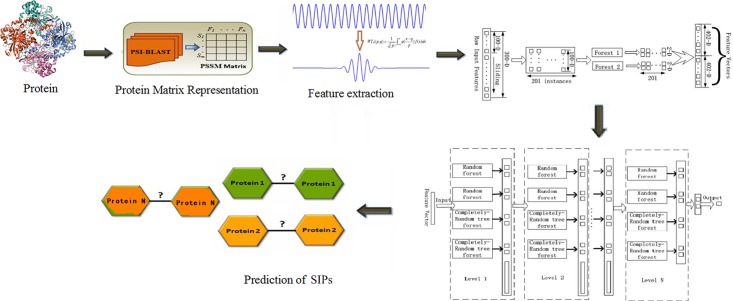
The flowchart of our work.

Given this potential, in this study we presented a novel approach for SIPs prediction, which combined deep forest with wavelet transform (WT) method based on PSSM of protein sequences. First, we widely collected the golden standard *human* and *yeast* datasets from common database, which can be integrated for discriminating SIPs. Second, Position-specific Iterative Basic Local Alignment Search Tool (PSI-BLAST) collated each protein sequence conversion for a PSSM. Third, WT approach was applied to calculate the feature values which could be input into deep forest, and then the SIPs prediction model was constructed. At last, we carried out experiments on the two golden standard datasets and compared the presented model with SVM method and other existing methods. Experimental results suggest that our proposed model works very well for SIPs prediction and can provide clues for understanding protein functions. We described our work as a Figure 1.

## Materials and Methods

### Datasets Preparation

In the experiment, we can derive 20,199 curated *human* protein sequences from the UniProt database (Consortium, 2014). Then, the PPI related information were integrated from all sorts of resources, including PDB (Berman et al., 2000), DIP (Salwinski et al., 2004), MINT (Licata et al., 2011), InnateDB (Breuer et al., 2012), IntAct (Orchard et al., 2013), BioGRID (Chatr-Aryamontri et al., 2017), and MatrixDB (Launay et al., 2014). The high quality data of these resources was sufficient for the creation of PPI prediction models. Here, we only paid close attention to those PPIs whose interaction types were labeled as “direct interaction” and for which the two interaction partners were identical. Finally, we can gather 2994 *human* self-interacting protein instances.

We need construct the golden standard datasets based on 2994 *human* SIPs mentioned above to measure the performance of the prediction model. It mainly includes the following steps (Liu et al., 2016): (1) We removed the protein sequences whose length <50 residues and >5000 residues from all the *human* proteome, because they may be fragments; (2) To construct *human* golden standard positive dataset, and ensure that the SIPs is of high quality. It must be meet one of the following requirements: 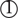 the protein has been announced as homo-oligomer (containing homodimer and homotrimer) in UniProt; 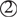 the self-interaction could be detected by more than one small-scale experiment or two large-scale experiments; 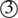 it has been reported by more than two publications for the self-interactions; (3) For *human* golden standard negative dataset construction, we removed the various kinds of SIPs from all the *human* proteome (including proteins characterized as “direct interaction” and more wide-ranging “physical association”) and the detected SIPs annotated in UniProt database. As a result, the ultimate *human* golden standard datasets consisted of 1441 SIPs and 15,938 non-SIPs. And then, the whole *human* datasets size is 17379.

According to the above-mentioned method, we also built the *yeast* golden standard datasets to further measure the cross-species capacity of our proposed model. Thus, the final *yeast* datasets contained 710 SIPs as positives and 5511 non-SIPs as negatives. And then, the whole *yeast* datasets size is 6221.

### Position Specific Scoring Matrix

In our achievements, position specific scoring matrix (PSSM) method is helpful to detect distantly related proteins (Gribskov et al., 1987; Gao et al., 2016; Wang L. et al., 2017;Wang Y.-B et al., 2017; Wang Y. et al., 2017). Accordingly, a PSSM was converted from each protein sequence information by employing the position specific iterated BLAST (PSI-BLAST) (Altschul and Koonin, 1998). And then, a given protein sequence can be transformed into an *H × 20* PSSM which can be announced as follow:

(1) M={Mαβ  α:1=1⋯H,β=1⋯20}

where the rows *H* of the matrix is the length of a protein sequence, and the columns represent the number of amino acids because of each protein gene was constructed by *20* types of amino acids. For the query protein sequence, the score *C*_αβ_ represents the β-*th* amino acid in the position of *α* which can be distributed from a PSSM. Thus, the score *C*_αβ_ can be defined as:

(2) $$C\alpha \beta =\sum_{k=1}^{20}p(\alpha ,k)\times q(\beta ,k)$$

where *p(α,k)* denotes the appearing frequency value of the *k-th* amino acid at position of *α* with the probe, and *q(β,k)* is the value of Dayhoff’s mutation matrix between β-*th* and *k-th* amino acids. Eventually, different fractions represent different positional relationships, a strongly conservative position can achieve a greater score, and otherwise a lower degree denotes a weakly conservative position.

In conclusion, PSSM have become essential to much research for predicting SIPs. Each PSSM from protein sequence was generated by PSI-BLAST algorithm, which can be employed for predicting SIPs. In a detailed and exact way, to get a high degree and a wide range of homologous sequences, the *E*-value parameter of PSI-BLAST was set to be 0.001 which reported for a given result represents the number of two sequences’ alignments and chose three iterations in this process. As a result, the PSSM can be denoted as a *20*-dimensional matrix which compose of *M × 20* elements, where the rows *M* of the matrix is the number of residues of a protein, and the columns of the matrix denote the *20* amino acids.

### Wavelet Transform

In signal processing, WT (Daubechies, 1990) is an ideal tool for signal time-frequency analysis and processing. The main point is that transformation can adequately highlight some aspects of the problems, and any details of signal can be focused. It solved the difficult problem of Fourier transform. And then, WT has been a major breakthrough in the scientific method since the Fourier transform.

In mathematics, WT is a new branch. It merges the technology of functional, Fourier analysis, harmonic analysis, and numerical analysis. A wavelet series is a representation of a square-integrable function by a certain orthonormal series generated by a wavelet. WT (Lewis and Knowles, 1992) was applied to decompose the image. WT also can be employed in many fields, such as signal processing (Sahambi et al., 1997), speech processing (Agbinya, 1996), and non-linear science (Staszewski, 1998). The main feature is that some characteristics of the problem can be fully highlighted by transformation, and then it can focus on any details of the problem.

The integral WT can be defined as follow:

(3) $$WT\varphi (p,q)=\frac{1}{\sqrt{\left|p\right|}}\int_{-\infty }^{\infty }\varphi (\frac{x-q}{p})f(x)dx$$

where, the binary dilation *p* = 2^-i^, and the dyadic position *q* = 2^-i^ *j*, and the wavelet coefficients were given by

(4) $$C_{\mathrm{ij}}=WT_{\varphi }(2^{-i},2^{-i}j)$$

And then, an orthonormal wavelet can be applied to define a function *φ𝜖L^2^(R)*. *L^2^(R)* is the Hilbert space. The Hilbert basis is built as the family of functions:

(5) $$\varphi_{\mathrm{ij}}(x)=2^{\frac{i}{2}}\varphi (2^{i}x-j)$$

where

(6) $$\left\{\varphi_{\mathrm{ij}}:i,j\in \mathbb{Z}\right\}$$

If under the standard inner product on *L^2^(R)*,

(7) $$\langle f,g\rangle =\int_{-\infty }^{\infty }f(x)\bar{g(x)}dx$$

which is orthonormal, this is an orthonormal system:

(8) $$\langle \varphi_{\mathrm{ij}},\varphi_{\mathrm{mn}}\rangle =\int_{-\infty }^{\infty }\varphi_{\mathrm{ij}}(x)\bar{\varphi_{\mathrm{mn}}(x)}dx=\delta_{\mathrm{im}}\delta_{\mathrm{jn}}$$

where δ_im_ is the Kronecker delta.

In order to satisfy the completeness that every function *f∈L^2^(R)* may be expanded in the basis as

(9) $$f(x)=\sum_{i,j=-\infty }^{\infty }C_{\mathrm{ij}}\varphi_{\mathrm{ij}}(x)$$

with convergence of the series understood to be convergence in norm.

However, the establishment of features extraction based on machine learning methods is a challenging mission. In bioinformatics and genomics, an amino acid sequence can be treated as a series of digital signals, and then, we can applied WT method to analyses them (Jia et al., 2016). Because each protein sequence contains different amount of amino acids which will bring about different length of feature vectors. We cannot directly transform a PSSM into a feature vector. Hence, we multiplied the transpose of PSSM by PSSM to get *20 × 20* matrix, and employed the feature extraction method of WT to generate feature vectors from the *20 × 20* matrix. Afterward, the eigenvalues of each protein sequence can be calculated as a *400*-dimensional vector. Eventually, each protein sequence of *yeast* and *human* datasets was converted into a *400*-dimensional vector by applying WT method.

In our research, in order to reduce the influence of unimportant information and increase the prediction accuracy, we used the PCA approach to remove noisy features from *yeast* and *human* datasets. So that we can reduce the dimension of the two datasets from 400 to 300. Furthermore, reducing the dimensionality of the datasets could use lower dimension of features to represent the main information, so as to speed up calculation speed.

### Deep Forest

As we all know, DNN have been successfully applied to various fields, such as visual and speech information (Hinton et al., 2012; Krizhevsky et al., 2012), leading to the hot wave of deep learning (Goodfellow et al., 2016; Chen and Huang, 2017; Chen et al., 2017). Zhou and Feng (2017) proposed deep forest, which also termed GCForest (multi-Grained Cascade Forest), that is a novel decision tree ensemble approach. Actually, it is used to do representation learning, which can find out the better features by end to end training. The performance of GCForest is more competitive than that of DNN.

**FIGURE 2:**
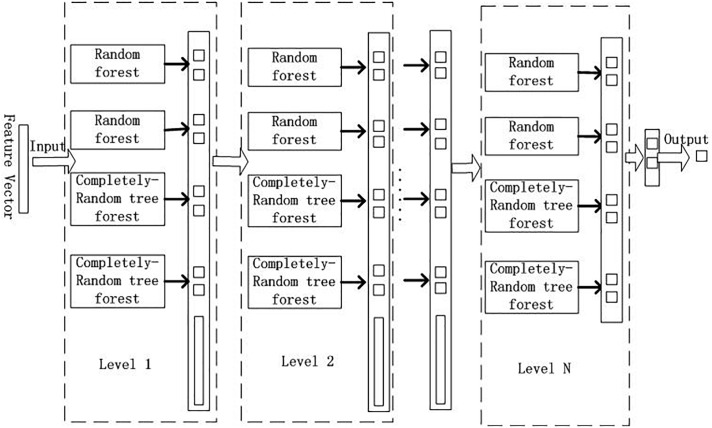
Cascade forest structure.

**FIGURE 3:**
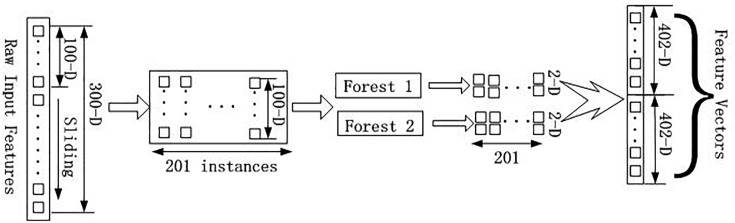
Flow chart of Multi-grained scanning approach.

GCForest model can deal with a wide variety of data from different domains, and whose training process has high computational efficiency and strong extensibility. In our experiment, the training process of GCForest model was mainly divided into two parts. The first part is devoted to the construction of cascade forest, as illustrated in Figure 2; The second part is multi-grained scanning, as shown in Figure 3.

From Figure 2, we input the feature vector which obtained by multi-grained scanning approach. GCForest employs a cascade structure, and each level of the cascade forest includes two random forests and two complete-random tree forests (Breiman, 2001). Each random forest contains 500 trees, and the *√d* number of features was chosen randomly as the candidate, and then the feature with the best *gini* value was selected as the segmentation. Each complete-random tree forest contains 500 complete-random trees, and the tree was generated by randomly choosing features to be partitioned at each node of the tree, and the tree grew until each leaf node only contains instances of the same class or no more than 10 instances. The number of trees in each forest was a hyper-parameter. It was a binary classification problem in our experiment, so the output of each forest will be a two-dimensional class vector, which is then linked to the input feature to represent the next original input. In order to reduce the risk of over-fitting, the class vectors generated by each forest are produced by k-fold cross validation.

From Figure 3, multi-grained scanning approach was applied to enhance the cascade forest. This method used sliding window to scan the raw input features which extracted from *human* and *yeast* datasets by WT method into our model, and then generate instances which was fed into forests to merge the new feature vectors. In our experiment, there are two classes, and the raw input features dimensions are 300, and the dimension of sliding window is100.

### Model Assessment

In order to intuitively present the availability and stability of our proposed model, in our study, we assessed our model and calculated the values of following parameters: Accuracy (Accu), specificity [Spec, also called true negative rate (TNR)], Precision [Prec, also named positive predictive value (PPV)], Recall [Sensitivity, also known as true positive rate (TPR)], F1_score (is the harmonic mean of precision and recall) and Matthews’s correlation coefficient (MCC), respectively. These parameters can be described as follows:

(10) $$Accu=\frac{TP+TN}{TP+FP+TN+FN}$$

(11) $$Spec=TNR=\frac{TN}{FP+TN}$$

(12) $$Prec=PPV=\frac{TP}{TP+FP}$$

(13) $$Recall=TPR=\frac{TP}{TP+TN}$$

(14) $$F1\_score=2\times \frac{Prec\times Recall}{Prec+Recall}=\frac{2TP}{2TP+FN+FP}$$

(15) $$MCC=\frac{\left(TP\times TN)-(FP\times FN\right)}{\sqrt{\left(TP+FN)\times (TN+FP)\times (TP+FP)\times (TN+FN\right)}}$$

where, *TP* represents the number of true positives, that is to say the count of true interacting pairs correctly predicted. *FP* represents the quantity of false positives, which defined as the count of true non-interacting pairs falsely predicted. *TN* represents the count of true negatives, which is the number of true non-interacting pairs predicted correctly. *FN* represents the quantity of false negatives, in other words, it represents true interacting pairs falsely predicted to be non-interacting pairs. On the basis of these parameters, we plotted a receiver operating curve (ROC) to assess the predictive properties and ability of our proposed model. And then, we can compute the area under curve (AUC) to evaluate the quality of the classifier.

## Results and Discussion

### Performance of GCForest on *Human* and *Yeast* Datasets

In order to illustrate that our proposed model can achieve good results as comprehensive as possible, we detected the *human* and *yeast* SIPs which was collected from multiple publicly available resources. In the experiment, we used cross validation to obtain reliable and stable model. Taking *human* dataset which was removed noisy features by PCA method as an example, the whole dataset was divided into five non-overlapping parts, and randomly selected four parts as training set, and the remaining part was taken as the independent test set. Next, to build the model on the training set, and evaluate the performance of the model on independent test set.

Based on the constructed data sets, we predicted the SIPs by using the proposed model. To ensure the fairness and objectivity of the experiment, the parameters of proposed model should be consistent on *human* and *yeast* datasets, respectively. The fewer hyper-parameters are contained in the GCForest model and the parameter setting is not very sensitive for the model. That is to say, GCForest model has high robustness for the hyper-parameters setting. But there are still some parameters need to be set up. In the experiment, we set shape_1X = 100 [shape of a single sample element (100, 100)], window = 100 (list of window sizes to use during Multi-Grain Scanning), tolerance = 5.0 (accuracy tolerance for the cascade growth).

Afterward, we implemented the proposed model on *human* and *yeast* datasets, respectively. The prediction results can be shown in Table 1. By cross-validation on the *human* and *yeast* datasets, we observed that the prediction accuracy of GCForest reached up to 95.43 and 93.65% on *human* and *yeast* datasets, respectively.

**Table 1:** Performance of proposed model on *human* and *yeast* dataset.

	Accu	Spec	Prec	Recall	F1_score	MCC
Datasets	(%)	(%)	(%)	(%)	(%)	(%)
*human*	95.43	99.09	84.07	54.06	65.81	65.26
*yeast*	93.65	99.28	88.73	47.01	61.46	61.87


As shown in Table 1 above, it is shown that the proposed model gained accuracy more than 93% for predicting SIPs on the two integrated datasets. We summed up that a reasonable classifier and feature extraction method is necessary and sufficient for SIPs prediction, and presented some reasons in the following: (1) The use of PSSM has greatly improved the prediction effect, which was transformed by PSI-BLAST. Not only can it describes the protein sequence in the terms of numerical forms, but also it contains useful enough information as much as possible. Accordingly, a PSSM provides almost all the major information of single protein sequence to detect SIPs. (2) The WT feature extraction method can find out more useful information of the protein sequences, and improve the performance of the prediction model. (3) GCForest is an appropriate classifier, and it can perform well when bound with the WT feature extraction method.

### Comparison of GCForest and SVM Method

As shown in section “ Datasets Preparation,” we can see that our proposed model can obtain a good performance on both *human* and *yeast* integrated datasets, respectively. But it is still necessary to further verify the effectiveness of the algorithm. In terms of classification, the state-of-the-art SVM is a common classification algorithm based on supervision learning model, which has been widely applied in a great deal of scientific research fields. Therefore, we compared the performance of GCForest with SVM classifiers to detect SIPs, employing the same features which extracted from the two integrated datasets described above. In the experiment, the LIBSVM packet tool (Chang and Lin, 2011) was mainly applied for classification. At the beginning of the experiment, we should set certain parameters of SVM. A radial basis function (RBF) was selected as the kernel function, and then using a grid search approach to adjust *c* and *g* of RBF, which were set up *c = 0.3* and *g = 1000*.

**FIGURE 4:**
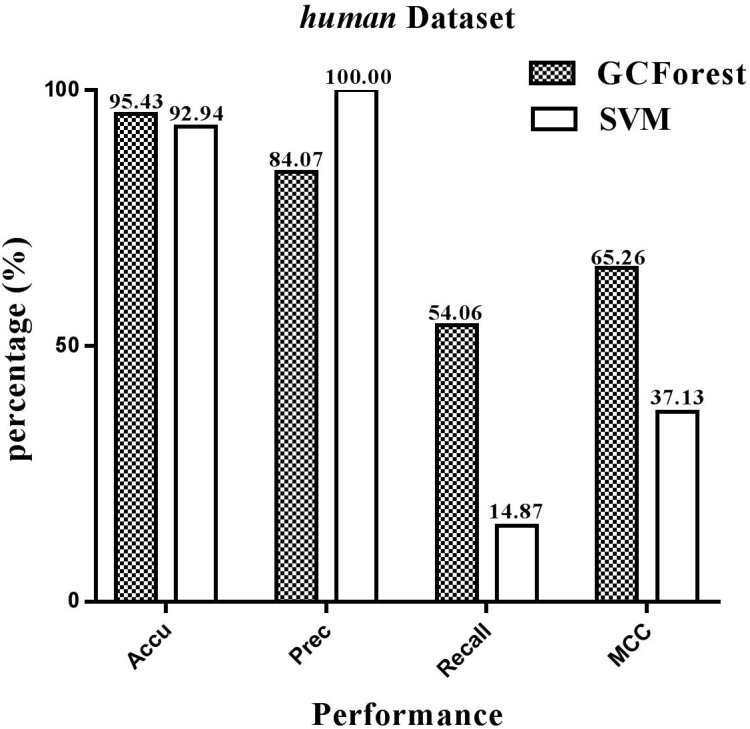
Performance between GCForest and SVM on *human* dataset.

**FIGURE 5:**
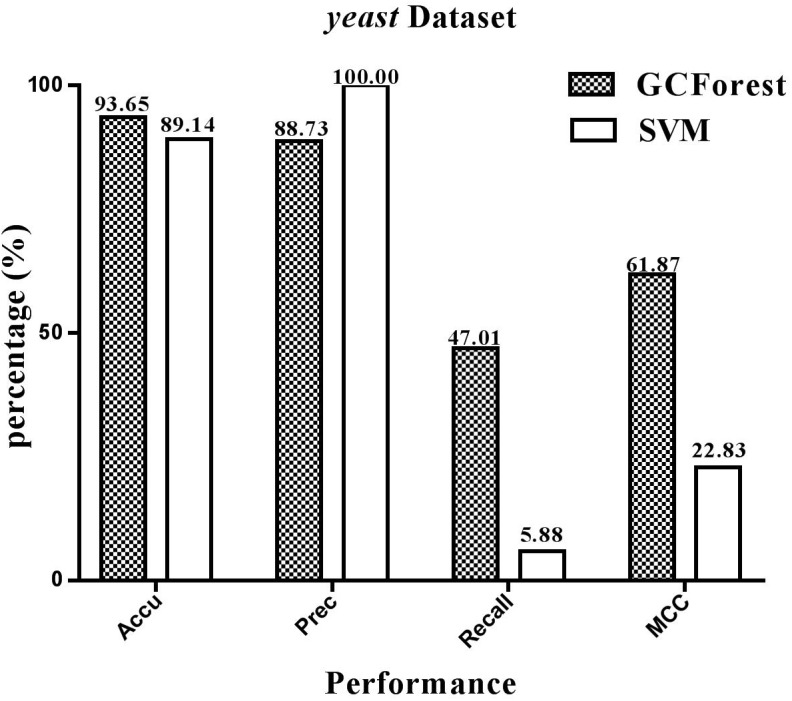
Performace between GCForest and SVM on *yeast* dataset.

The performance statistics reported in Figure 4, 5 were obtained comparing the proposed model and SVM-based model on *human* and *yeast* datasets, respectively. From Figure 4, on the *human* dataset, the prediction accuracy for both GCForest and SVM classifier were greater than 92%; the precision was 84.07% (GCForest) and 100% (SVM); the recall was 54.06% (GCForest) and 14.87% (SVM); the MCC was 65.26% (GCForest) and 37.13% (SVM). From Figure 5, the accuracy, the precision, the recall, and the MCC of SVM classifier are 89.14, 100.00, 5.88, and 22.83% on the *yeast* dataset; Nevertheless, the GCForest classifier achieved 93.65% accuracy, 88.73% precision, 47.01% recall, and 61.87% MCC. These results all suggest that our proposed model is superior to those of SVM-based approach, and it has comparable performance in SIPs prediction.

### Compare GCForest With Other Existing Methods

To further illustrate that our GCForest model has higher prediction ability, we also measured the performance of our proposed model with other existing methods based on *human* and *yeast* datasets, respectively. As shown in Tables 2, 3, we listed a clear statement of account that the accuracy of GCForest model was higher than that of other existing methods on the two integrated datasets (mentioned in section “Materials and Methods”). The same as Spe, MCC, and F1 Score. However, the recall (also named sensitivity, the true positive rate) of proposed model was lower than that of other existing methods, which measures the percentage of true positives that are successfully identified as having the condition. The reason may be that traditional PPI predictor could not work well for predicting SIPs because of the utilized correlation information between two proteins, such as co-localization, co-expression and co-evolution. These results on *human* and *yeast* datasets all indicate that our proposed model was justified to be a better deep learning method to detect SIPs in this work.

**Table 2:** Measure the quality of GCForest and the other methods on human dataset.

	Accu	Spec	Recall	MCC	F1 Score
Model	(%)	(%)	(%)	(%)	(%)
SLIPPER (Chang and Lin, 2011)	91.10	95.06	47.26	41.97	46.82
DXECPPI (Du et al., 2014)	30.90	25.83	87.08	8.25	17.28
PPIevo (Zahiri et al., 2013)	78.04	25.82	87.83	20.82	27.73
LocFuse (Zahiri et al., 2014)	80.66	80.50	50.83	20.26	27.65
CRS (Liu et al., 2016)	91.54	96.72	34.17	36.33	36.83
SPAR (Liu et al., 2016)	92.09	97.40	33.33	38.36	41.13
Random forest	94.33	100.00	29.14	52.39	45.13
**Proposed method**	**95.43**	**99.09**	**54.06**	**65.26**	**65.81**


**Table 3:** Measure the quality of GCForest and the other methods on yeast dataset.

	Accu	Spec	Recall	MCC	F1 Score
Model	(%)	(%)	(%)	(%)	(%)
SLIPPER (Chang and Lin, 2011)	71.90	72.18	69.72	28.42	36.16
DXECPPI (Du et al., 2014)	87.46	94.93	29.44	28.25	34.89
PPIevo (Zahiri et al., 2013)	66.28	87.46	60.14	18.01	28.92
LocFuse (Zahiri et al., 2014)	66.66	68.10	55.49	15.77	27.53
CRS (Liu et al., 2016)	72.69	74.37	59.58	23.68	33.05
SPAR (Liu et al., 2016)	76.96	80.02	53.24	24.84	34.54
Random Forest	92.77	100.00	44.10	63.81	61.21
**Proposed method**	**93.65**	**99.28**	**47.01**	**61.87**	**61.46**


### Receiver Operating Characteristic (ROC) Curve

The ROC curve, also called sensitivity curve, was widely used a great deal of fields such as medicine, bioinformatics, forecasting natural hazards, model performance assessment and so on. It is a comprehensive index reflecting the continuous variables of sensitivity and specificity, and it is a method to reveal the relationship between sensitivity and specificity. According to a series of different binary classification methods, the curve was plot with false positive rate (FPR, also called sensitivity) as abscissa and true positive rate (TPR, also named 1-specificity) as ordinate. We also used ROC curve to analysis the performance of the prediction model.

**FIGURE 6:**
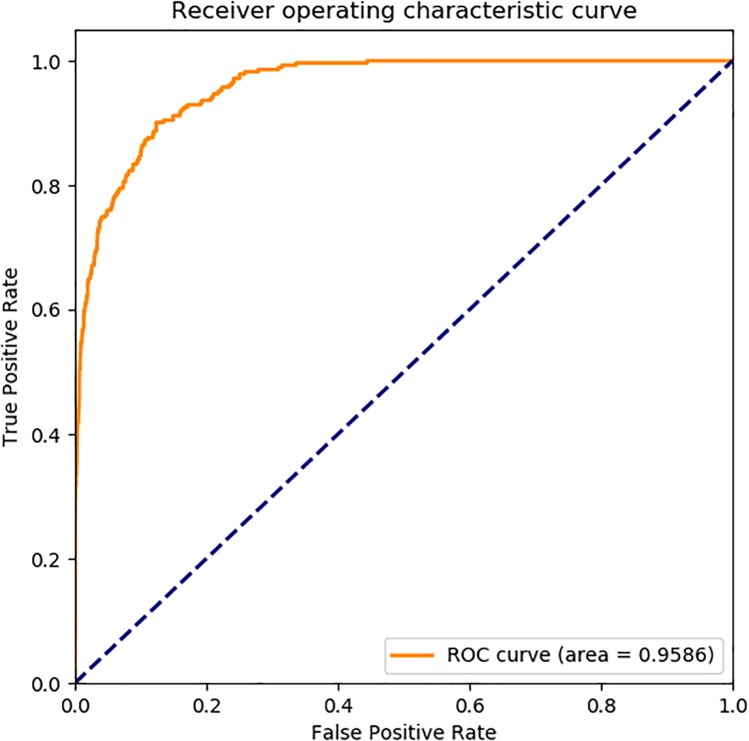
ROC curve of GCForest based on the results of *human* SIPs dataset.

**FIGURE 7:**
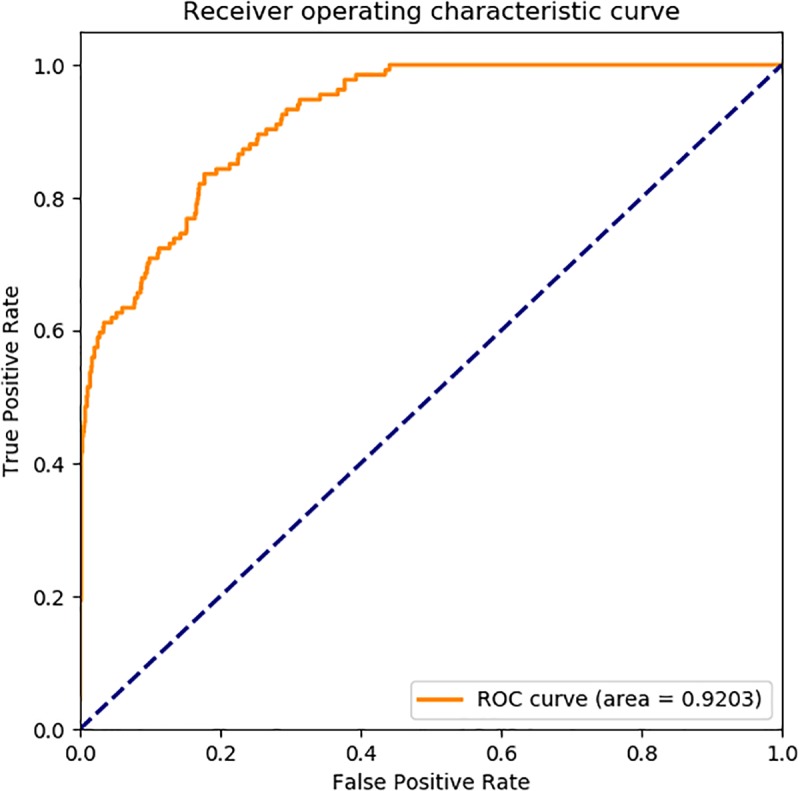
ROC curve of GCForest based on the results of *yeast* SIPs dataset.

In Figure 6, the ROC curve of our presented model performed on *human* SIPs dataset, it is shown that the AUC is 0.9586. The ROC curve of put forward model assessed on *yeast* SIPs dataset is shown in Figure 7, it is clear that the AUC is 0.9203. Therefore, the proposed model is necessary and sufficient for SIPs detection.

## Conclusion

In this study, we developed an improved deep learning-based model that was applied to predict whether an identified protein is likely to interact or not. More specifically, firstly, we converted the PSSM turned from each protein sequence into a 400-dimensional feature vector by employing the WT feature extraction method; then, in order to decrease the influence of noise and remove the redundant information, we reduced the dimension of the feature vector to 300 by using PCA dimensional-reduced method; finally, realized classification on *human* and *yeast* datasets by applying GCForest model. The performance of the proposed model achieved an accuracy of 95.43 and 93.65% on the *human* and *yeast* golden standard datasets, respectively. It is revealed that our model is suitable and perform well for detecting SIPs. We also compared it with SVM-based and other popular existing method, and the comparison empirical results show that the proposed model is superior to the SVM-based methods and other previous methods. It is anticipated that our proposed model can act as a potential tool in the SIPs prediction research.

## Data Availability

The datasets for this manuscript are not publicly available because the data is too big to share. Requests to access the datasets should be directed to chenzhanheng17@mails.ucas.ac.cn.

## Author Contributions

Z-HC and L-PL conceived the algorithm, carried out the analyses and experiments, prepared the data sets, and wrote the manuscript. J-RZ, ZH, and LW designed, performed and analyzed experiments, and wrote the manuscript. All authors read and approved the final manuscript.

## Conflict of Interest Statement

The authors declare that the research was conducted in the absence of any commercial or financial relationships that could be construed as a potential conflict of interest.
